# The TGFβ1 Promoter SNP C-509T and Food Sensitization Promote Esophageal Remodeling in Pediatric Eosinophilic Esophagitis

**DOI:** 10.1371/journal.pone.0144651

**Published:** 2015-12-14

**Authors:** Renee Rawson, Arjun Anilkumar, Robert O. Newbury, Vineet Bafna, Melissa Aquino, Jacob Palmquist, Hal M. Hoffman, James L. Mueller, Ranjan Dohil, David H . Broide, Seema S. Aceves

**Affiliations:** 1 Division of Allergy, Immunology, University of California San Diego, La Jolla, California, United States of America; 2 Center for Infection, Immunity, and Inflammation, University of California San Diego, La Jolla, California, United States of America; 3 Division of Pediatric Gastroenterology and Nutrition, University of California San Diego, La Jolla, California, Rady Children’s Hospital, San Diego, California, United States of America; 4 Department of Pediatrics, University of California San Diego, La Jolla, California, Rady Children’s Hospital, San Diego, California, United States of America; 5 Department of Medicine, University of California San Diego, La Jolla, California, United States of America; 6 Department of Pathology, University of California San Diego, La Jolla, California, Rady Children’s Hospital, San Diego, California, United States of America; 7 Department of Computer Science and Engineering, University of California, San Diego, La Jolla, California; Harvard Medical School, UNITED STATES

## Abstract

**Background:**

Eosinophilic esophagitis (EoE) is a chronic antigen mediated disease associated with substantial esophageal remodeling and fibrosis. The functional TGFβ1 promoter SNP C-509 associates with renal fibrosis and asthma. The effect of TGFβ1 genotype and EoE severity or potential gene-environment interactions have not been previously reported in EoE.

**Methods:**

Genotype at TGFβ1 C-509T and remodeling was analyzed in 144 subjects with EoE. The severity of remodeling and inflammation was analyzed in the context of IgE sensitization to food antigens and C-509T genotype.

**Results:**

The TGFβ1 promoter C-509 genotypes CC, CT, and TT were 35%, 52%, and 13%, respectively. Sixty-six percent of subjects were sensitized to foods by positive skin prick test (SPT) or serum specific IgE. TT genotype subjects had significantly more TGFβ1 (CC subjects = 1300 per mm^2^; TT = 2250 per mm^2^) (p<0.05) and tryptase (CC subjects = 145 per mm^2^: TT = 307 per mm^2^) (p<0.05) positive cells and higher epithelial remodeling scores (2.4 vs 3.7, p<0.001) than CC subjects. The differences in TGFβ1 and tryptase positive cells as well as fibrosis were significantly increased when there was concurrent food sensitization. Food sensitization alone did not associate with any parameters of inflammation or remodeling.

**Conclusions:**

Our data support a gene-environment interaction between food and genotype at C-509 that modulates disease severity in EoE. Since EoE subjects often continue to consume foods to which they are sensitized, these findings may have clinical relevance for disease management.

## Introduction

Eosinophilic esophagitis (EoE) is a chronic, food antigen mediated disease of increasing worldwide prevalence [1]. Complications include esophageal rigidity and dysmotility with resultant dysphagia, food impactions, and strictures [2–7]. Esophageal remodeling consists of fibrosis, angiogenesis, and smooth muscle hypertrophy and is believed to be the underlying mechanism for disease complications [8–11]. The risk factors, reversibility, and rate of progression of esophageal remodeling are not entirely clear [11–14] but these issues are of significant clinical importance if we hope to halt or reverse remodeling.

The majority of untreated EoE subjects progress to esophageal narrowing with resultant complications such as food impactions [6,7]. Risk factors for food impactions include decreased use of topical corticosteroids [15]. On the genetic level, it has been reported that subjects who respond to EoE therapy have up-regulation of FKBP51 [16]. We have also reported that children with TGFβ1 promoter genotype CC at -509 respond better to therapy than those with CT or TT genotype [12]. Genetic risk factors for EoE include polymorphisms in thymic stromal lymphopoetin (TSLP), its receptor (TSLPR), the tissue specific gene calpain 14, the trafficking protein ANKRD21 as well as genes associated with atopic and autoimmune diseases, specifically c11orf30 and STAT6 [17,18].

Despite these findings, the genes that control disease severity in EoE and potential gene-environment interactions are not clear. In the esophagus, food can be considered an environmental trigger in EoE. The majority of pediatric EoE patients have IgE sensitization to multiple foods [1,19]. However, the clinical significance of this is not clear although food allergy has been associated with elevated iNKT cells and younger patients and the high affinity IgE receptor is present in EoE biopsies [20–22]. Recent reports suggest that the use of serum food specific IgE may be of assistance when creating elimination diets [23]. In contrast, multiple institutions have reported that skin prick testing is not useful in isolation when assessing EoE triggers [19,24,25]. There have been limited reports that, in EoE, IgE sensitization associates with increased esophageal mastocytosis [26]. Unlike patients with clinical IgE mediated food hypersensitivity, EoE patients often continue to consume the foods to which they are sensitized due to clinical tolerance. As such, the esophagus is routinely exposed to foods that could cause local mast cell degranulation, increased TGFβ1, and fibrosis.

In this study, we assess the potential interaction between the functional TGFβ1 promoter SNP C-509T, food sensitization, and EoE severity. We hypothesized that, since children with EoE continue to consume the foods to which they are sensitized, but not allergic and since TGFβ1 C-509T associates with fibrotic severity in other diseases, there would be worsening EoE severity with food sensitization. Herein we report for the first time that food sensitization in the context of C-509T associates with more severe mastocytosis and fibrosis in patients who have a CT or TT genotype. These data may have implications for EoE management.

## Methods

### EoE subjects/biopsies/staining

EoE subjects treated during routine clinical care and upper endoscopy (esophagogastroduodenoscopy, EGD) with biopsy at the UCSD/RCHSD eosinophilic gastrointestinal disorders clinic were recruited for genetics and database studies. EoE was defined as ≥15 eosinophils per high power field (hpf) on hematoxylin/eosin (H&E) stain at 400x magnification on light microscopy in the presence of typical symptom and endoscopic features. Serum or skin prick testing (SPT) for foods was performed as part of routine clinical care. Positive serum testing was defined as >0.35 kU/L. Positive SPT was defined as 3mm wheal and 5mm flare larger than the saline control. Paraffin embedded biopsy specimens were analyzed for fibrosis, TGFβ1, tryptase, and pSmad2/3 positive cells, and vWF and VCAM-1 positive blood vessels. 155 total patients were genotyped and 144 biopsies had adequate tissue for staining and/or LP for remodeling analysis (S1 Table). All research involving human participants has been approved by the University of California, San Diego/Rady Children's Hospital, San Diego Institutional Review Board (IRB). Written informed consent/assent was obtained from the participants (study numbers 091485 and 081415).

H&E stained, formalin fixed, paraffin embedded specimens were scored by a single pathologist blinded to the diagnosis and treatment (RN). The numbers of epithelial and lamina propria (LP) eosinophils, the severity of basal zone hyperplasia, and the LP fibrosis score were quantified using our previously published pathology scoring tool. [27]

Tissue sections (5μM) were deparaffanized and hydrated prior to immunostaining as previously described [8]. The mean of the peak numbers of TGFβ1, pSmad2/3, and tryptase positive cells and vWF/VCAM positive vessel per mm^2^ in 3 hpf are reported. Images were quantified and analyzed under identical light or fluorescence microscopic conditions, including magnification, gain, camera position, and background illumination.

### Analysis of the C-509T SNP of the TGFβ1 promoter

DNA was isolated from peripheral blood and C-509 genotype was analyzed using Taqman based PCR analysis according the manufacturer’s instructions (Applied biosystems for SNP rs1800469). Genotype was confirmed in a subset of subjects using direct sequencing (forward—CAGACTCTAGAGACTGTCAG, reverse-GTCACCAGAGAAAGAGGAC) and analysis (Sequencher, Genecodes, Ann Arbor MI).

### Statistics

Between group comparisons were done using NCSS and Graphpad Prism statistical software and groups were compared using an ANOVA test for multiple groups or t test for 2 groups. A p value <0.05 was considered statistically significant. Hardy-Weinberg equilibrium was tested using the a two-tailed HWExact test in the Hardy Weinberg R package.

## Results

### Clinical characteristics

One hundred and fifty-five EoE subjects were genotyped at promoter SNP C-509 and 144 had adequate tissue for analysis of remodeling features. Of these subjects, 35% were genotype CC, 52% were CT, and 13% were TT. A two-tailed Hardy-Weinberg Exact test showed that Hardy-Weinberg equilibrium could not be rejected for this data (p = 0.13). The mean age of the subjects was 4.9 years and 55% had histologic failure on PPI monotherapy. Eighty-one percent of the subjects were male and 65% were Caucasian. Consistent with high rates of atopy in the EoE population, 34% had asthma, 61% had allergic rhinitis, 27% had eczema, and 45% had self-reported food allergy. Clinical characteristics between genotypes are shown in Table 1.

**Table 1:** Clinical characteristics by genotype.

Genotype	Age (years)	Caucasian (%)	Male (%)	Aeroallergen Positive % (Total N)	Food Positive % (Total N)
CC	8	84	88	75(16)	73(40)
CT	6.4	84	84	70(50)	62(63)
TT	5.7	67	53	56(9)	69(13)

Sixty-six percent had food positive IgE to one or more foods by serum or skin prick testing. The most common EoE triggers in children and adults are milk, wheat, egg, and soy. In this population, 77% of the children had serum specific IgE to egg, milk, wheat and/or soy; 63% had serum IgE to milk, 52% to wheat, 43% to egg, and 37% to soy. The mean levels of IgE were 4.4ku/L for milk, 6.8ku/L for wheat, 6.1kU/L for egg, and 8.4kU/L for soy. In order to assess both the presence and the function of IgE, we analyzed the children who were SPT positive to foods. Thirty-two percent of children were SPT to milk, 42% to egg, 23% to wheat, and 22% to soy.

### Inflammation and remodeling by genotype

**Fig 1:**
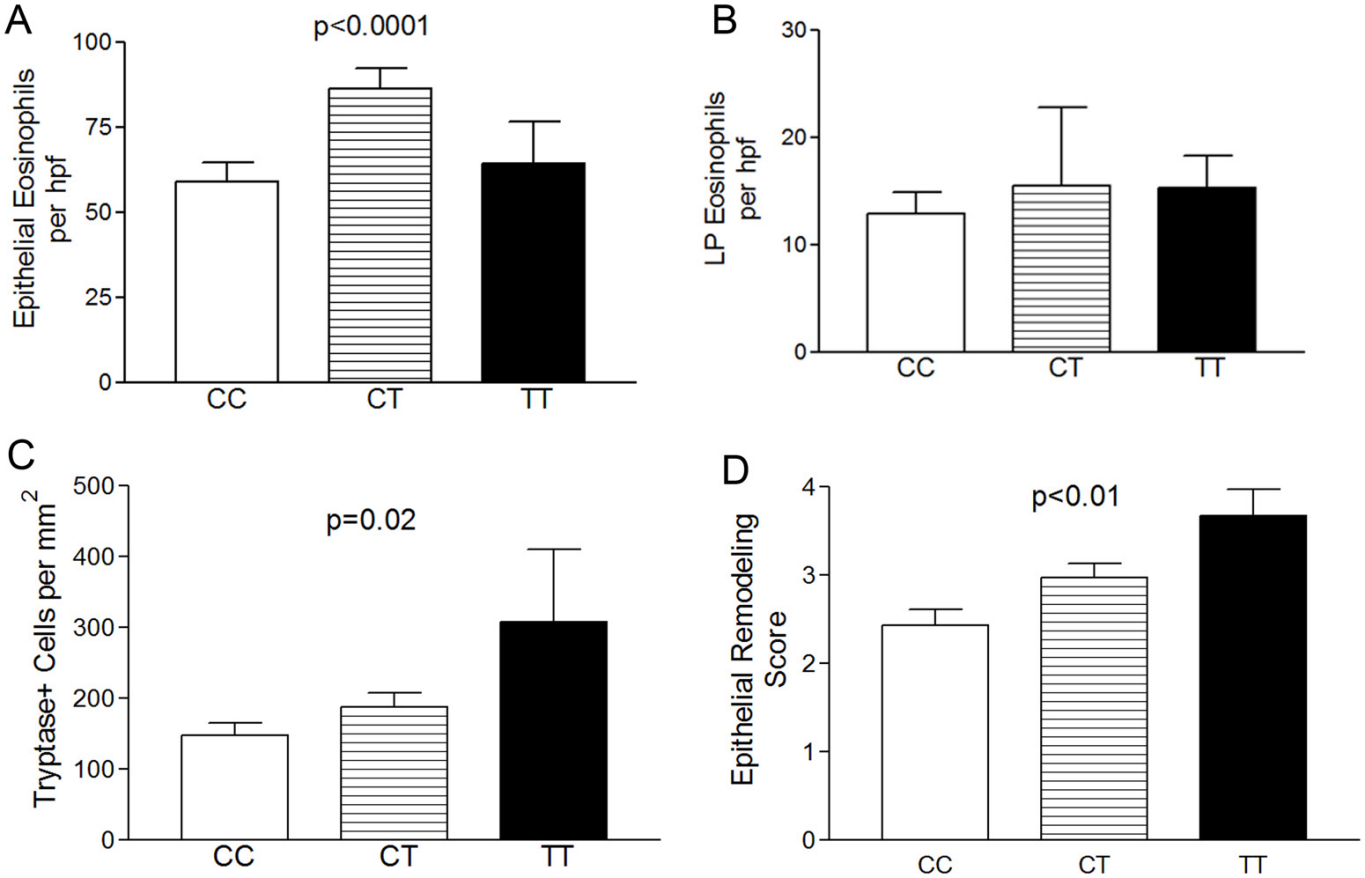
Inflammatory cells and epithelial remodeling by genotype. CT genotype subjects have the highest numbers of epithelial (A) and lamina propria (LP) eosinophils (B) but TT genotype subjects have the highest numbers of tryptase positive mast cells (C) and epithelial remodeling (D). Bars represent mean with standard error.

Subject biopsies were assessed during the active disease state. One hundred and forty (97%) of subjects were not on any EoE directed therapy at the evaluated biopsy. Four patients (3%) had active EoE despite treatment with topical fluticasone (2) or budesonide (2) (3 CC, 1 CT, mean eosinophils per hpf = 69). The mean number of epithelial eosinophils per hpf were higher in CT genotype (86±10) patients as compared with CC (54±7), (p<0.05) and TT (86±27) (p = 0.10) (Fig 1a). By contrast, there was no significant difference in the numbers of LP eosinophils by genotype alone (Fig 1b). There were significantly more tryptase positive mast cells per mm^2^ in subjects TT genotype subjects as compared with CC (p = 0.01) (Fig 1c). We calculated an epithelial remodeling score which we define as the severity of basal zone hyperplasia + presence of dilated intercellular spaces + presence of epithelial desquamation. There was a stepwise increase in epithelial remodeling from CC to TT genotype (Fig 1d). (Fig 1 Inflammatory cells and epithelial remodeling by genotype).

**Fig 2:**
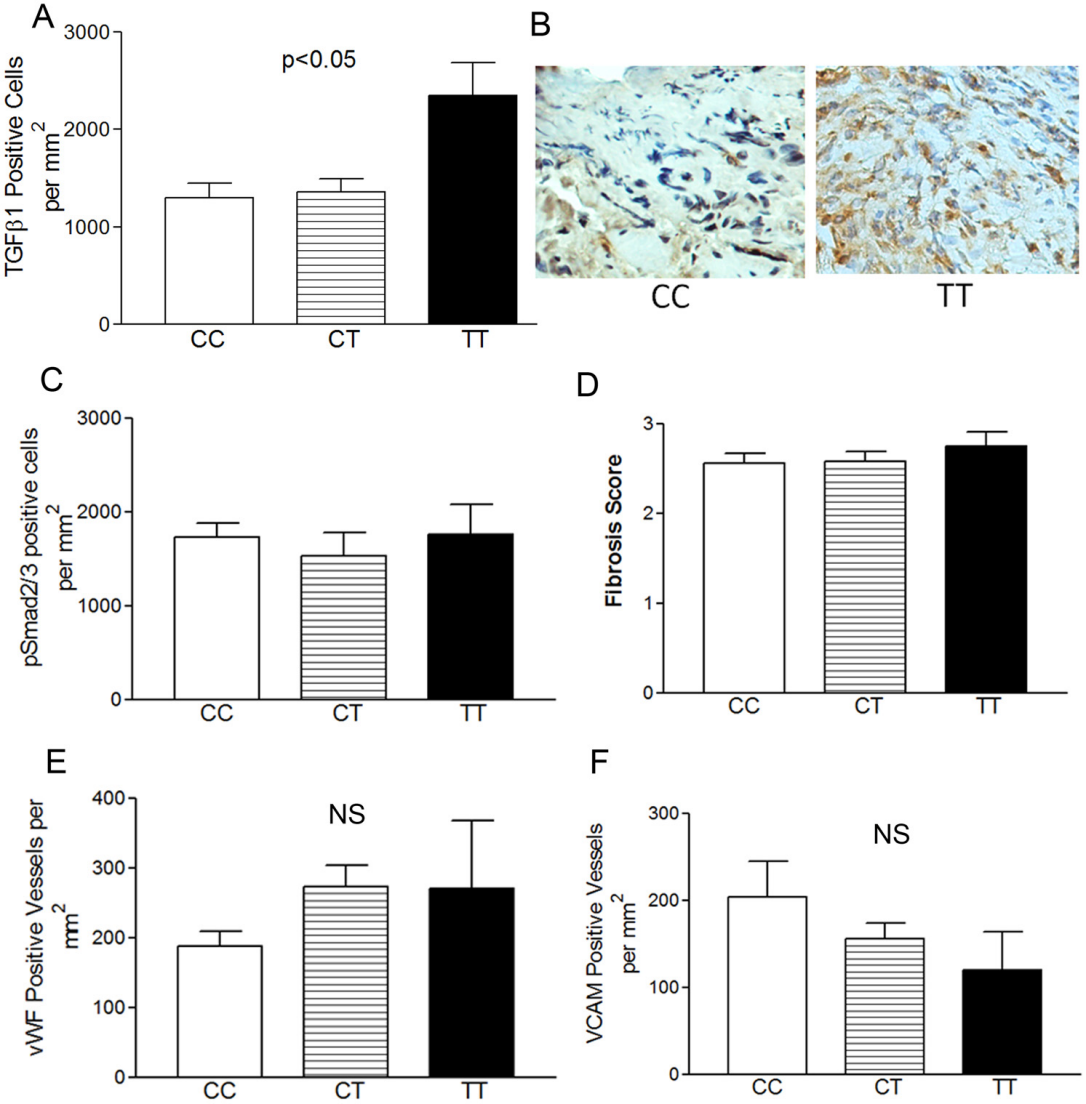
Lamina propria remodeling features by genotype. TT subjects have the highest numbers of TGFβ1 positive cells (A, B). Representative image of TGFβ1 positive cells in TT versus CC genotype subjects (B). pSmad2/3 positive cells by genotype (C), fibrosis score by genotype (D), and vWF (E) and VCAM (F) positive vessels by genotype.

It has been reported that the TT SNP at C-509 creates a transcription factor binding site that does not exist in CC subjects thereby increasing TGFβ1 gene transcription. TT genotype can associate with more severe disease [28–30]. We assessed if the numbers of TGFβ1 positive cells by genotype. There was a significant difference by between genotypes (CC = 1300 vs CT = 1434 vs TT = 2350 TGFβ1 positive cells per mm^2^) (p = 0.009) (Fig 2a and 2b). There were no differences in the numbers of pSmad2/3 positive cells (canonical TGFβ1 signaling) by genotype (Fig 2c). While there appeared to be a higher fibrosis scores in TT as compared with CC or CT subjects, this was not significant (Fig 2d). We also analyzed the degree of angiogenesis and vascular activation between genotypes and found no significant differences (Fig 2e and 2f). We calculated EGD scores based on endoscopic features of lichenification, pallor, plaques, furrows, rings/strictures, and friability. Though these tended to be higher in TT subjects, there were no statistical differences between the groups (not shown) (Fig 2 Lamina propria remodeling features by genotype). Since the TT population had a lower percentage of Caucasian subjects, we analyzed the histologic features by Caucasian versus non-Caucasians and found that there were no differences in any histologic or molecular features by sub-population (fibrosis score, TGFβ1+ cells, tryptase+ cells, epithelial eosinophils, and epithelial remodeling score all p>0.3).

### Inflammation and remodeling by genotype in the context of food sensitization

**Fig 3:**
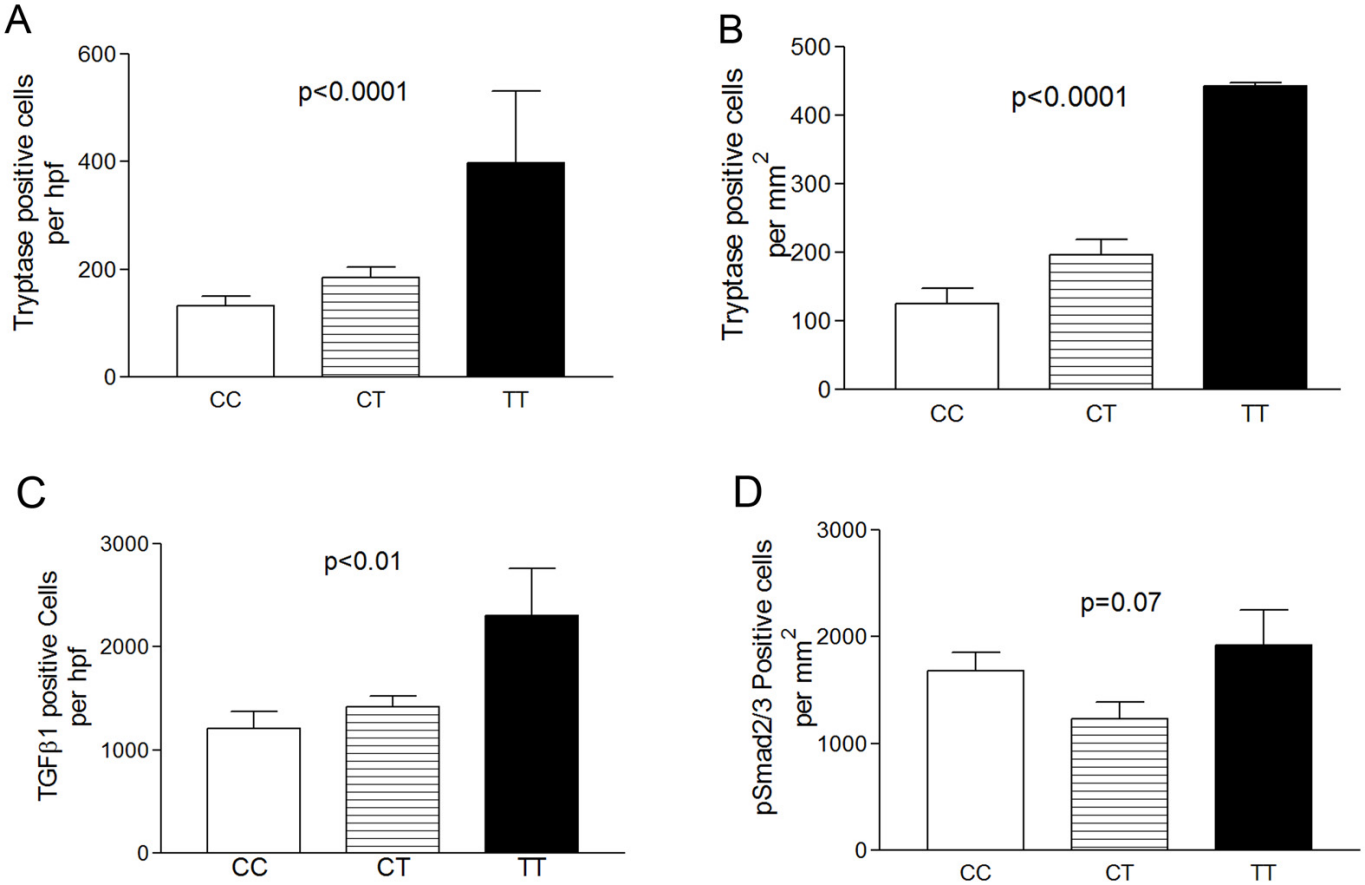
Mast cells and remodeling parameters in subjects by genotype with concurrent food sensitization. Tryptase positive cells by genotype in subjects with serum and/or skin prick positive food specific IgE (A) or food sensitization only on skin prick testing (B). TGFβ1 (C) and pSmad2/3 (D) positive cells and vWF positive blood vessels (E) by genotype with coexistent food sensitization

In order to understand if food sensitization could influence the degree of inflammatory or remodeling severity, we analyzed these parameters by genotype in the food sensitized subgroup of subjects. Food sensitization was defined as food specific IgE on serum or skin testing. Eosinophilic inflammation was not altered in the presence of food sensitization in the epithelium or LP (data not shown). However, consistent with the fact that food specific IgE could affect mast cells, we saw significantly fewer tryptase positive mucosal mast cells in CC (132 per mm^2^) subjects as compared with both CT (184 per mm^2^, p = 0.06) or TT subjects (398 per mm^2^ p = 0.001 for CC versus TT and 0.005 for CT versus TT) in the food sensitized group (Fig 3a). This difference by genotype was even more pronounced in the group of subjects who had food SPT positivity (Fig 3b). When comparing only within a genotype (rather than across genotypes), only TT genotype subjects had more tryptase positive cells in those subjects who were food SPT positive (578±334) as compared with subjects who were food SPT negative (124±88), but this did not reach statistical significance. (Fig 3 Mast cells and remodeling parameters in subjects by genotype with concurrent food sensitization).

Food sensitization affected remodeling in the context of genotype. The differences in TGFβ1 positive cells between genotypes was more pronounced with coexistent food sensitization (Fig 3c), and pSmad2/3 positive cells tended to be higher in TT versus CT subjects (p = 0.07) (Fig 3d). Vascular activation with VCAM-1 remained unchanged by food sensitization but the numbers of vWF positive blood vessels per mm^2^ was significantly higher in CT (280±45) as opposed to CC (174±24) subjects (p = 0.04) (Fig 3d and 3e).

**Fig 4:**
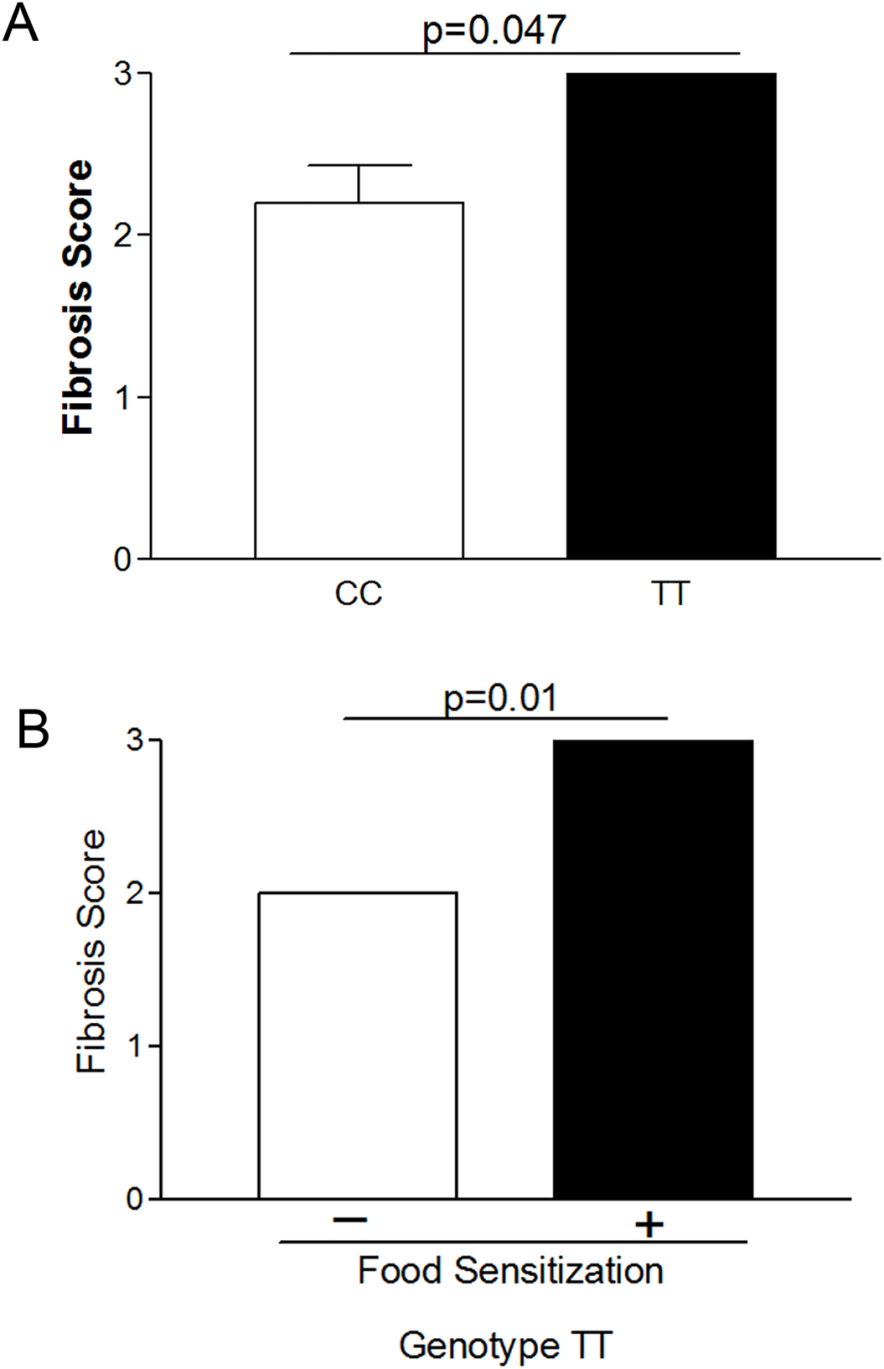
Genotype TT subjects have higher fibrosis. Fibrosis score by genotype among subjects with skin prick test positivity (A) and within the TT genotype between subjects with and without food sensitization (B)

Fibrosis was affected by food sensitization plus genotype. Food SPT positive TT genotype patients had significantly higher fibrosis scores than CC subjects with positive SPT (Fig 4a). In addition, within TT genotype subjects, those with positive food SPT had significantly higher fibrosis scores despite the small numbers of subjects (p = 0.01) (Fig 4b). (Fig 4 Genotype TT subjects have higher fibrosis)

## Discussion

In this study we demonstrate a number of novel findings in pediatric EoE subjects. We show the first potential gene-environment interaction in pediatric EoE between the TGFβ1 SNP C-509 and food antigen sensitization. This functional promoter SNP in the TGFβ1 gene has been shown to cause a binding site for the transcription factor YY-1 thereby increasing TGFβ1 gene transcription [30] which could lead to increased fibrosis. Herein we report that subjects with genotype TT have significantly more TGFβ1 expressing cells than those who are genotype CC or CT. This increase in TGFβ1 occurred independently of food sensitization, demonstrating that the TT genotype at C-509 could be a pro-fibrotic risk factor. The rates of TT genotype do not differ between EoE and the CEU control population and there are reasonable incidence of this SNP in all major sub-populations (http://www.snpedia.com/index.php/Rs1800469) which aligns with our hypothesis that C-509T is not a risk factor for EoE per se, but rather, a disease modifying allele.

Mast cells are produce of TGFβ1 in EoE. TGFβ1 increases pro-fibrotic gene expression and induces esophageal smooth muscle cell contraction [31,32]. We found that there was a step-wise gradient of mast cell numbers by genotype with TT genotype being the highest in the context of SPT positivity to foods. This could have important implications in disease management, since foods could induce local mast cell degranulation with TGFβ1 release and subsequent fibrosis and smooth muscle contraction. Since both mast cells and basophils are tryptase positive, it is also possible that basophils comprise a subset of the tryptase positive cells. As such, it may be of utility to avoid foods in subjects with severe disease and/or genotype TT and/or who do not respond well to other EoE therapies. Interestingly, a recent study has demonstrated successful EoE therapy using serum food specific IgE based elimination diet which was equivalent in success to empiric six food elimination diet [23]. However, this is in contrast to isolated SPT based testing that is not of utility when creating an elimination diet [19,24,33].

We also demonstrate that a gene-environment interaction may influence fibrotic severity. TT genotype occurs in 10% of the general population, the same as in EoE subjects, demonstrating that unlike TSLP and its receptor, TGFβ1 C-509 seems not a likely genetic risk factor for EoE but a disease modifying gene [17]. This is consistent with other fibrotic diseases such as asthma and renal fibrosis where C-509 can affect disease risk and severity and our prior data in small cohorts that showed the patients with CC genotype were more likely to respond to topical corticosteroids [12,34–36]. TT subjects had higher fibrosis scores when there was SPT positivity to foods. This discovery is intriguing since it suggests that function and not merely presence of specific IgE could influence disease severity in the context of TGFβ1 genotype. Certainly, other environmental disease modifiers, such as distance of the residence from a freeway and house dust mite positivity, have been shown to change asthma in the context of C-509 genotype [29,37]. Our rates of food sensitization align with those seen in other allergic disorders such atopic dermatitis and children with a history consistent with food allergy or referred for food allergic reactions [38,39].

Children with EoE often continue to consume the foods to which they are sensitized, since substantial IgE sensitization without anaphylaxis is common in EoE. Although not all of our subjects failed PPI at/prior to EoE diagnosis, the current literature suggests that PPI-responsive esophageal eosinophilia is a clinical and molecular phenotype of classic PPI-resistant EoE [40]. Since our data suggest that food sensitization could influence disease severity, it will be interesting to evaluate if those children who are of CT and/or TT genotype and do not respond topical corticosteroids have higher rates of food sensitization. A recent publication demonstrated that among asthmatic subjects with genotype TT, there is a higher risk for loss of control of clinical disease [41]. In this context, it will also be interesting to understand if the T allele in EoE associates with those subjects who lose long-term control of their disease despite adherence to prescribed medications and/or diets. This is particularly important since EoE is a chronic disease with very high relapse rates upon removal of the disease-controlling therapy. Perhaps food eliminations based on SPT positivity could be an accessory tool for EoE management in subjects who relapse despite continued topical corticosteroid treatment.

Our study does have limitations. Although we were able to assess 144 children, this is a small sample size for the rare TT genotype. In addition, as a single center study, we may not have the phenotypic variation seen in multicenter studies. Half of our patients failed PPI monotherapy, leaving a possibility of some PPI-responsive esophageal eosinophilia among our population and potentially suggesting that the interaction between food sensitization and TGFβ1 genotype at C-509 may extend beyond allergen driven EoE. Lastly, as a largely Caucasian, male disease, we do not have robust genetic signals for other ethnicities. However, the large proportion of white males may help to diminish some genetic heterogeneity in our sample.

The role of TGFβ1 in allergic diseases is complex. However, it does appear that higher levels of TGFβ1 and/or TGFβ1 signaling associate with a more severe allergic phenotype. For example, people with Loewy’s-Dietz syndrome caused by increased signaling through the TGFβ1 receptor have higher rates of food allergy [42] and connective tissue disorders such as Marfan’s syndrome in which there is also increase TGFβ1 signaling, associates with EoE [43]. All of this would suggest that increased TGFβ1 can predispose to a more severe allergic phenotype.

In conclusion, our current data support a model in which the genotype at the functional promoter SNP C-509 can influence EoE severity, especially in the context of food sensitization. It will be of interest to confirm these findings in a larger cohort of subjects and to understand other influences that C-509 may have in the EoE esophagus.

## Supporting Information

S1 Table Cells and remodeling features by genotype. The numbers of eosinophils, mast cells, TGFβ1 positive, SMAD positive cells, vWF and VCAM positive vessels, fibrosis score, epithelial remodeling score, and presence/absence of food sensitization by genotype.
(PDF)
