# Supplementary material for: The TGFβ1 Promoter SNP C-509T and Food Sensitization Promote Esophageal Remodeling in Pediatric Eosinophilic Esophagitis
Source: PLoS One. 2015 Dec 14;10(12):e0144651. doi: 10.1371/journal.pone.0144651 (PMC4678166; doi:10.1371/journal.pone.0144651)
Supplement: S1 Table — The numbers of eosinophils, mast cells, TGFβ1 positive, SMAD positive cells, vWF and VCAM positive vessels, fibrosis score, epithelial remodeling score, and presence/absence of food sensitization by genotype. (PDF) [file pone.0144651.s001.pdf]

Supplemental Table

Genotype	Epithelial Eos per hpf *	Epithelial Remodeling Score	Fibrosis Score	Tryptase+ Cells	TGFb1 + Cells	SMAD+ Cells	VWF+ Vessels	VCAM+ Vessels	Food Sensitized**
CC	67.00	2.00	1	248	983	1336	119		Yes
CC	22.00	4.00	3	67	1330	1587	134	124	Yes
CC	105.00	4.00		239					Yes
CC	53.00	0.00			710	3581			Yes
CC	17.00	1.00	3	157	464	1650	92	32	Yes
CC	24.00	3.00	3	89				41	Yes
CC	48.00	4.00		72	745	2101	168	148	Yes
CC	46.00	4.00	3	132	1317	780	137	789	No
CC	108.00	1.00		260	2437	1296	42	250	Yes
CC	46.00	1.00	3	92	1736	1041	205	178	No
CC	46.00	3.00	2	216	465	248	74		Yes
CC	84.00	4.00		125	1463	2583	99	287	Yes
CC	86.00	1.00	2	183					No
CC	34.00	3.00	1	139	2216	1979	136	102	Yes
CC	75.00	2.00	2	316	2032	1710	147	51	Yes
CC	66.00	4.00	2	142	1650	1462	81	135	No
CC	29.00	1.00		214	867	1852	55	0	Yes
CC	52.00	3.00	2	161	1097				Yes
CC	84.00	5.00	3	123	650	607	201		Yes
CC	21.00	3.00	2	52	775	1459	156		Yes
CC	37.00		2	213					ND
CC	85.00	4.00	3	72	878	1011	38	112	Yes
CC	50.00	3.00	3	132	851	1047	88	369	Yes
CC	79.00	3.00	3	116	1122	1023	104	127	Yes
CC	88.00	1.00	1	146	2007		193		Yes
CC	10.00	3.00	3			635	92	149	ND
CC	34.00	1.00	3	83	1831	1602	84		No
CC	31.00	1.00							ND
CC	22.00								ND
CC	50.00	3.00		646	3305	3019	444		ND
CC	2.00	1.00			4230		417		Yes
CC	11.00	1.00		11	764		410	344	Yes
CC	44.00	4.00		299	566	1646	211		Yes
CC	89.00	3.00	3	0	602	1945	320	149	Yes
CC	24.00	2.00		74					Yes
CC	90.00	0.00	2	52	907	2271	299		Yes
CC	75.00	1.00		16					Yes
CC	45.00	4.00	3	64	377	2909	341	192	ND
CC	21.00	3.00	3	9	492		365	552	Yes
CC	155.00	4.00		199		2299			ND
CC	56.00	2.00		139	1834	3471	428		No
CC	17.00	3.00		126					Yes
CC	52.00	1.00		37					ND
CC	84.00	2.00							No
CC	65.00	2.00		173					No
CC	238.00	4.00	3	288	1519	2738	140	158	Yes
CC	65.00	3.00		117	668	1017	190		No
CC	66.00	3.00	3						ND
CC	102.00	3.00	3						ND
CC	57.00	1.00	3						ND
CC	20.00	0.00	2						No
CC	17.00	2.00	3						ND
CC	31.00	2.00	3						ND
CC	50.00	3.00	3						No
CC	16.00	3.00	3						No
CT	215.00	3.00		87					Yes
CT	78.00	2.00	1	319	1605	1601	179	178	Yes
CT	115.00	3.00	3	224	1651	1314	440	360	Yes
CT	50.00	5.00	3	77	1573	858	141	162	Yes
CT	33.00	1.00	3	170	946	1598	184	18	Yes
CT	56.00	3.00	3	207	1358	1811	553		Yes
CT	110.00		3	137	1386	532	328	131	ND
CT	70.00	4.00	3	130	1416	1202	364	186	ND
CT	27.00	3.00	3		535	1746		200	No
CT	105.00	4.00	3		1443				No
CT	145.00	2.00	3	222					Yes
CT	122.00	4.00		54					Yes
CT	4.00	4.00	3		2093				Yes
CT	66.00	3.00	3	154	1667	1412	187	94	ND
CT	127.00	1.00	3	35	1288	343	102		Yes
CT	89.00	1.00		33		2024	200		No
CT	105.00	4.00	3	321	1187	1878	117	148	No
CT	85.00	5.00	3	276	1134	833			Yes
CT	70.00	4.00	3	96	1881	511			Yes
CT	89.00	3.00	3	78	1416	898		55	No
CT	67.00	4.00	3	182	1333	583	118		Yes
CT	104.00	2.00		161	1564	1801	74	66	No
CT	126.00	3.00	0	127	764	1646			ND
CT	138.00			203	1494	874	146	90	No
CT	118.00	3.00	3	338					No
CT	32.00	4.00		129					ND
CT	105.00	4.00	3	144	1918	2296	93	167	Yes
CT	90.00	2.00	2	85	2086				Yes
CT	120.00	3.00		113	1355	451		98	Yes
CT	64.00	3.00		193	1509	802	262	82	No
CT	136.00	4.00		182		1408			Yes
CT	78.00	0.00	1	148	1364	1127	168	140	ND
CT	130.00	2.00	2		1997	460		268	Yes
CT	230.00	2.00		392					Yes
CT	125.00	4.00	2	444	1546	264			No
CT	60.00	1.00			1266				Yes
CT	71.00	4.00	2	353	1533	957	269	98	Yes
CT	158.00	4.00	3	236	481	390	158	50	Yes
CT	130.00		3	283	1428	544	69	110	No
CT	65.00	4.00		118	1316	973	143	62	Yes
CT	114.00	1.00	3	130	1261	462	146		Yes
CT	105.00	5.00		159	1337	1834	198	31	No
CT	42.00	3.00	2	391	2736	3314	216		Yes
CT	30.00	1.00							Yes

CT	85.00	4.00	3	583	2104	1225	252	283	Yes
CT	70.00	4.00	1	224				81	Yes
CT	23.00			110					No
CT	5.00	0.00		649					No
CT	81.00	2.00	2	218	1094	478	131	234	Yes
CT	74.00	3.00	1	116	1927	1361	130	222	Yes
CT	205.00	3.00		294	1446	1257	61		ND
CT		0.00	1		3707	3604		209	ND
CT	42.00		3	109					No
CT	33.00	1.00		50		1168	437		Yes
CT	93.00	3.00		83	6741		434		ND
CT	55.00			204					Yes
CT	95.00	2.00	3		289		621		ND
CT	35.00	4.00	3	129	446	545	360	333	Yes
CT	60.00	2.00	2	118	556	829	488	431	ND
CT	125.00	4.00	3			11564	146	303	No
CT	82.00	3.00		167	1062		677		Yes
CT	91.00	4.00	3	158	205		358		No
CT	19.00	1.00	na	893					No
CT	214.00	5.00		76					ND
CT	90.00	2.00	3	19	963	1699	335	234	No
CT	55.00	4.00	3	102	866	3296	462	312	Yes
CT	21.00	3.00	2	12	821	6705	393		No
CT	136.00	4.00		139					Yes
CT	49.00	4.00	3	6	465	2031	110	70	Yes
CT	68.00	5.00	3	141	69	1591	602	242	ND
CT	275.00	1.00		0		1619	775		No
CT	19.00	2.00	3	193					Yes
CT	175.00	4.00	3	231	921	1751	870		Yes
CT	29.00	3.00							No
CT	49.00	1.00		14					No
CT	29.00	5.00	3	254		NO LP	NO LP	NO LP	ND
CT	125.00	4.00	3			NO LP	NO LP	NO LP	Yes
CT	110.00	3.00	3						No
CT	88.00	4.00	3						No
CT	17.00	4.00	3						ND
TT	55.00	4.00	3	321	2284	1823	78	41	Yes
TT	120.00	3.00	2	252	4490	2170	176	80	Yes
TT	75.00	4.00		189					ND
TT	155.00	3.00	3	321	2998	3127	140	33	Yes
TT	35.00	4.00			3151				No
TT	27.00	4.00		28					No
TT	23.00	3.00	3	159	2611	852	141	201	Yes
TT	45.00	2.00		73	2460	788			ND
TT	68.00	7.00							Yes
TT	59.00	5.00							Yes
TT	18.00	5.00	3	348	1662	1255	104	246	Yes
TT	30.00	3.00			1764		761		No
TT	75.00	3.00	3						Yes
TT	61.00	5.00	3		1111		492		Yes
TT	58.00	4.00		204	964	2299			Yes
TT	214.00			1183					Yes
TT									Yes
TT	30.00	2.00							ND
TT	26.00	3.00	2						No
TT	15.00	2.00							No

*If eosinophils per hpf (high power field) <15, a level not used had >15

**Sensitized= Positive on serum or skin prick testing
